# Guava pomace: a new source of anti-inflammatory and analgesic bioactives

**DOI:** 10.1186/1472-6882-13-235

**Published:** 2013-09-24

**Authors:** Carina Denny, Priscilla S Melo, Marcelo Franchin, Adna P Massarioli, Keityane B Bergamaschi, Severino M de Alencar, Pedro L Rosalen

**Affiliations:** 1 Department of Physiological Sciences, Piracicaba Dental School, University of Campinas – UNICAMP, P.O. Box 52, 13414-018 Piracicaba, SP, Brazil; 2 Department of Agri-food Industry, Food and Nutrition, "Luiz de Queiroz" College of Agriculture, University of São Paulo, Piracicaba, SP, Brazil

**Keywords:** Agro-industrial residue, Anti-inflammatory, Antinociceptive, Quercetin, Epicatechin, Guava pomace

## Abstract

**Background:**

Guava pomace is an example of the processing waste generated after the manufacturing process from the juice industry that could be a source of bioactives. Thus, the present investigation was carried out in order to evaluate the anti-inflammatory and antinociceptive potential and determinate the main phenolic compounds of a guava pomace extract (GPE).

**Methods:**

The anti-inflammatory activity was evaluated by carrageenan, dextran, serotonin, histamine-induced paw edema and neutrophils migration in the peritoneal cavity models. Acetic acid-induced abdominal writhing and formalin test were performed to investigate the antinociceptive effects. In addition, the content of total phenolic and of individual phenolic compounds was determined by GC/MS.

**Results:**

GPE showed anti-inflammatory activity by carrageenan, dextran, serotonin, histamine-induced paw edema and neutrophils migration in the peritoneal cavity models (p < 0.05). GPE also demonstrated antinociceptive activity by acetic acid-induced abdominal writhing and formalin test (p < 0.05). The total phenolic value was 3.40 ± 0.09 mg GAE/g and epicatechin, quercetin, myricetin, isovanilic and gallic acids were identified by GC/MS analysis.

**Conclusions:**

The presence of bioactive phenolic compounds as well as important effects demonstrated in animal models suggest that guava pomace could be an interesting source of anti-inflammatory and analgesic substances.

## Background

*Psidium guajava*, usually known as guava, is an important tropical fruit mostly consumed fresh. The Guava industry provides a variety of processed products, such as beverages, syrup, ice-cream, jams, jellies, toffee, juice, and dehydrated and canned products. Since the worldwide production of guava is estimated at about 500,000 metric tons, considerable amounts of waste from this industry are also generated and simply discarded to the environment [1].

A great variety of agro-industrial residues from many species of fruits are wasted every year, polluting the environment [2,3]. Efforts have been made to use residues to generate several value-added products, such as bioactive substances, used by food, cosmetic and pharmaceutical industries [3,4]. Guava pomace is an example of the processing waste generated after the manufacturing process and represents up to 15% of the original fruit [1].

In addition to being an import food crop, guava is an important medicinal plant that has been traditionally used for a long time in countries of the tropical America [1,5]. This species is commonly used to treat gastrointestinal and respiratory disturbances and as an anti-inflammatory. Several different studies have been developed to support its popular use [5,6]. Revolving around its anti-inflammatory and analgesic properties, most studies refer to the leaf extracts, which have been evaluated on several experimental models [5,7-9]. A preliminary study about the anti-inflammatory and antinociceptive activity of guava fruits was reported by Sen *et al*. [7]. Despite this, the precise effects of *P. guajava* and the signaling pathways involved remain unknown. In general, biological properties of guava have been already associated with its phenolic compounds, such as protocatechuic, ferulic, ascorbic, gallic and caffeic acids and quercetin [5].

Concerning the guava pomace, this solid agro-industrial residue consists of a mixture of peel, seed and pulp that is rich in phenolic compounds with antioxidant capacity [2]. Therefore, due to the great potential demonstrated in the literature through studies that identified important bioactive compounds, either in the fruit, leaves or the pomace, the purpose of the present study was to determine the anti-inflammatory and antinociceptive potential by different *in vivo* models, as well as the total phenolic content, and the main constituents by GC/MS of the guava pomace extract.

## Methods

### Plant material

The pomace from the processing of guava (*Psidium guajava* L. *-* Myrtaceae) was provided by "Cepêra - Agro Industrial Ibitirama Ltda", a food company located in the City of Monte Alto, SP, Brazil, in March 2009. The material (1745.08 g) was lyophilized, homogenized, weighed and stored at -18°C.

### Preparation of the extract

The air-dried and powdered guava pomace (100 g) was extracted with 160 mL of ethanol (EtOH) and 40 mL of water (H_2_O) by using an ultrasound for 30 minutes (3 times). The obtained guava pomace extract (GPE) was filtered and evaporated using a rotary evaporator and freeze dryer to provide the crude dried extract. The dried extract was stored at -18°C until its use.

### Determination of the total phenolic content

The total phenolic content was determined by the Folin-Ciocalteu method [10]. The analysis was performed following the spectrophotometric method, using Folin-Ciocalteau’s reagent (Dinâmica Química Contemporânea, Diadema, SP, Brasil) and gallic acid (Sigma-Aldrich, St. Louis, MO, USA) as standard.

A volume of 0.5 mL of the extract and 2.5 mL of Folin-Ciocalteu’s reagent (diluted in water 1:10) was placed in tubes and, after five minutes, 2 mL of sodium carbonate (4%) were added. The tubes were kept away from the light and, after two hours, the absorbance was read in a spectrophotometer (Shimadzu, Kyoto, Japan) at 740 nm. The total phenolic content was expressed as mg of gallic acid equivalent (GAE) per g of extract (mg GAE/g).

### Gas chromatography with mass spectrometry (GC-MS)

GPE was purified with solid phase extraction (SPE DSC-18 Discovery, 2 g, Supleco, Sigma-Aldrich, St. Louis, MO, EUA) and 100 μL of N-methyl-N-(trimethylsilyl) trifluoroacetamide (MSTFA) were added for derivatization. The silanized sample was analyzed in a gas chromatograph (Shimadzu GC 2010) coupled with a mass spectrometer (Shimadzu QP 2010 Plus) and equipped with a capillary column (30 m × RTX 5MS 0.25 mm × 0.25 μm). The programming temperature started at 80°C (1 min) at a heating rate of 20°C min^-1^ and reached 250°C (1 min), going to to 300°C (5 min) at a rate of 6°C min^-1^, 310°C (5 min) at a rate of 15°C min^-1^ and 320°C (10 min) at a rate of 20°C min^-1^, at a total of 40 minutes of analysis. Helium was used as the carrier gas. The injector temperature was 280°C and an injection volume of 0.5 μL was used in splitless mode. The interface was maintained at 280°C and the detector operated in scanning mode (m/z 40–800) [11]. Phenolic compounds were identified by comparison with the data obtained from GC/MS (retention time and fragmentation ion) of Extrasynthese Co. authentic standards (syringic acid, myricetin, kaempferol, luteolin, liquiritigenin, isoliquiritigenin, quercetin, *p*-coumaric acid, ferulic acid, catechin, epicatechin) and with the Wiley 8 library. The results were presented as means and followed by the standard deviation.

### Animals

Male Balb/c albino mice (20–25 g), SPF, were purchased from CEMIB/UNICAMP (Multidisciplinary Center for Biological Research, SP, Brazil) and used as experimental animals. The mice were maintained in a room with controlled temperature (22 ± 2°C) for a 12 h light/12 h dark cycle, humidity (40-60%), with food (standard pellet diet) and water provided *ad libitum*. The experiments were conducted in accordance with the Guide for the Care and Use of Laboratory Animals and counted on the prior approval from the local Animal Ethics Committee (CEUA, Ethics Committee on Animal Use/UNICAMP, process number 2155–1).

### Carrageenan-induced paw edema

The method by Winter *et al*., [12] was followed. A paw edema was induced by subplantar injection of 0.05 mL of lambda carrageenan (1% w/v in 0.9% of saline) into the left hind-paw in mice. An equal volume of vehicle was injected into the contralateral paw. The volume of both hind-paws up to the ankle joint was measured with a plethysmometer (model 7140, Ugo Basile) immediately before (0), 1, 2, 3, 4 and 5 hours after carrageenan. The difference in the volumes between the hind-paws was a measure of the edema (mL). The GPE (30, 100, 300, 1000 mg/kg), the reference drug, indomethacin (10 mg/kg), or the vehicle (10 mL/kg of 0.9% of saline), were given intraperitoneally 1/2 h or orally 1 h before the subplantar injection of the phlogistic agent.

### Dextran, histamine and serotonin induced paw edema

The anti-inflammatory activity of the extract was tested with three phlogistic agents (dextran, histamine and serotonin). The paw edema was induced in mice by subplantar injection of freshly prepared dextran (100 μg/0.05 mL), histamine (50 μg/0.05 mL) or serotonin (1 μg/0.05 mL) in 0.9% NaCl solutions, respectively. The paw volume was recorded at 0 and 1 h after injecting histamine or serotonin and 0, 1 and 2 h after injecting dextran. GPE (30, 100 and 300 mg/kg), cyproheptadine (2 mg/kg) and vehicle (10 mL/kg, NaCl 0.9%) were intraperitoneally administered 1/2 h before eliciting the paw edema.

### Neutrophils migration in the peritoneal cavity

In order to determine the neutrophil migration to the peritoneal cavity GPE (30, 100 and 300 mg/kg) or Indomethacin (10 mg/kg) were administered by subcutaneous (s.c.) injection, 30 min before the administration of inflammatory stimuli by intraperitoneal (i.p.) injection of carrageenan at 500 μg/cavity. The vehicle (0.9% NaCl) was used as negative control. Mice were killed 4 h after the challenge (carrageenan) administration and the peritoneal cavity cells were harvested by washing the cavity with 3 mL of phosphate buffered saline (PBS) containing EDTA. The volumes recovered were similar in all experimental groups and equal to approximately 95% of the injected volume. In order to count the total number of cells, a Newbauer chamber was used. Smears were prepared using a cytocentrifuge (Cytospin 3; Shandon Lipshaw), stained with Panotic staining kit and the different cells were counted (until 100 cells) using an optical microscope (1000 ×). The results are presented as the number of neutrophils per cavity.

### Acetic acid-induced abdominal writhing

The total number of writhes following the intraperitoneal administration of 0.2 mL of 1% (v/v) acetic acid was recorded over a period of 30 min, starting 5 min after the acetic acid injection. The animals were pretreated with GPE (30, 100 and 300 mg/kg, i.p.), vehicle (0.9% NaCl, i.p.), or indomethacin (10 mg/kg, i.p.), 20 min before administering the acetic acid [13,14].

### Formalin test

The method used in the present study was similar to that previously described by [15]. The animals were treated with GPE (30, 100 and 300 mg/kg, i.p.), 30 min before injection under the surface of the right hind paw of 25 μL of 2.5% formalin (0.92% formaldehyde) in saline. Indomethacin (10 mg/kg, i.p.) and morphine (5 mg/kg, i.p.) were used as the positive control, and vehicle (0.9% NaCl, i.p.) was used as the negative one. Animals were observed from 0–5 min (neurogenic phase) and 15–30 min (inflammatory phase) and the time spent licking the injected paw was recorded with a chronometer and considered as indicative of nociception**.**

### Statistical analysis

Data are expressed as the mean ± standard error of the mean (SEM). Statistical comparisons between groups were made using variance analyses (ANOVA) followed by Tukey’s or Dunnett’s tests (GraphPad Prism for Windows, Version 5.0). The significance was accepted when the p value was ≤ 0.05.

## Results and discussion

GPE was primarily tested for anti-inflammatory effects using a carrageenan-induced hind-paw edema model as a classic *in vivo* activity screening model. Carrageenan-induced paw edema is found to be biphasic, the initial phase is due to the release of histamine, serotonin and bradykinin in the first hour after the administration of carrageenan, and a more pronounced second phase is attributed to the release of prostaglandin, bradykinin, protease, and lysosome-like substances within 2–3 h [12]. The dose-dependent swelling thickness and inhibition effects of the extract are presented in Table 1. Indomethacin (10 mg/kg, i.p.) was used as positive control in this assay, and it showed maximum inhibition (70%) after 3 hours. The extract prepared from guava pomace administrated intraperitoneally showed inhibition (p < 0.05) with the 300 mg/kg dose after the 1st, 2nd and 3rd hours (46, 56 and 38%, respectively). At a dose of 100 mg/kg, the extract inhibited 51 and 42% at the 1st and 2nd hours, respectively (p < 0.05). There was no significant difference in the inhibition of paw edema between the extract at a dose of 30 mg/kg and the control group. When administrated orally, the extract at a dose of 1000 mg/kg also inhibited paw edema (Table 2).

**Table 1:** Effect of i.p. administration of guava pomace extract (GPE) on a carrageenan-induced paw edema

**Groups**	**Dose (mg/kg)**	**Mean Edema ∆V mL (percent inhibition)**				
		**1 h**	**2 h**	**3 h**	**4 h**	**5 h**
Control	-	0.10 ± 0.01	0.12 ± 0.01	0.14 ± 0.01	0.15 ± 0.01	0.09 ± 0.01
Indo	10	0.03 ± 0.01*	0.03 ± 0.01**	0.04 ± 0.01**	0.07 ± 0.01*	0.08 ± 0.01
		(62)	(68)	(70)	(48)	(10)
GPE	300	0.05 ± 0.01*	0.05 ± 0.01*	0.08 ± 0.00*	0.09 ± 0.01	0.05 ± 0.01
		(46)	(56)	(38)	(35)	(42)
GPE	100	0.05 ± 0.01*	0.08 ± 0.01*	0.10 ± 0.01	0.11 ± 0.01	0.10 ± 0.01
		(51)	(42)	(27)	(4)	(13)
GPE	30	0.06 ± 0.01	0.07 ± 0.01	0.11 ± 0.01	0.09 ± 0.01	0.11 ± 0.03
		(41)	(40)	(20)	(39)	(-)

∆V = the values represent the mean difference of paw volume between the basal measure and others measures (1, 2, 3, 4 and 5 h) ± S.E.M.; n = 5-6. *p < 0.05, **p < 0.01, significantly different when compared to control (ANOVA followed by Dunnett’s test).

**Table 2:** Effect of p.o administration of guava pomace extract (GPE) on a carrageenan-induced paw edema

**Groups**	**Dose (mg/kg)**	**Mean Edema ∆V mL (percent inhibition)**				
		**1 h**	**2 h**	**3 h**	**4 h**	**5 h**
Control	-	0.10 ± 0.00	0.14 ± 0.01	0.16 ± 0.01	0.18 ± 0.01	0.16 ± 0.01
Indo	10	0.07 ± 0.01	0.09 ± 0.01	0.09 ± 0.01*	0.09 ± 0.01*	0.09 ± 0.01*
		(30)	(30)	(49)	(67)	(61)
GPE	1000	0.06 ± 0.01	0.06 ± 0.01*	0.06 ± 0.01	0.06 ± 0.01	0.06 ± 0.01
		(35)	(42)	(20)	(19)	(04)

∆V = the values represent the mean difference of paw volume between the basal measure and others measures (1, 2, 3, 4 and 5 h) ± S.E.M.; n = 5-6. *p < 0.05, significantly different when compared to control (ANOVA followed by Dunnett’s test).

From the carrageenan paw edema results, since the extract showed higher activity in the first hours, guava pomace extract was subjected to the paw edema induced by dextran. This phlogistic agent once administered into the animal paw is able to promote the consequent release of histamine and serotonin from mast cells. Non-steroidal anti-inflammatory agents are not able to inhibit this kind of inflammatory process [16]. Moreover, histamine and serotonin-induced paw edema were also used to confirm the potential involvement of these mediators with the anti-edematogenic effect observed. Thus, the extract (300 mg/kg) showed significant anti-inflammatory effects also on dextran-induced paw edema model that can be compared with the standard drug cyproheptadine (p < 0.05). However, there was no significant difference of inhibition of dextran paw edema between extract (100 and 30 mg/kg) and the control group (Table 3). Furthermore, at doses of 300 and 100 mg/kg, the extract showed significant anti-inflammatory effects on the histamine or serotonin-induced paw edema model (p < 0.05) (Table 4).

**Table 3:** Effect of i.p. administration of guava pomace extract (GPE) on a dextran-induced paw edema

**Groups**	**Dose (mg/kg)**	**Mean Edema (∆V mL)**	
		**1 h**	**2 h**
Control		0.12 ± 0.02	0.10 ± 0.04
Cyproheptadine	2	0.04 ± 0.02***	0.05 ± 0.03*
GPE	300	0.03 ± 0.02***	0.03 ± 0.02**
GPE	100	0.08 ± 0.02	0.07 ± 0.02
GPE	30	0.09 ± 0.02	0.15 ± 0.04

∆V = the values represent the mean difference of paw volume between the basal measure and others measures (1 and 2 h) ± S.E.M.; n = 5-6. *p < 0.05, **p < 0.01, ***p < 0.001, significantly different when compared to control (ANOVA followed by Dunnett’s test).

**Table 4:** Effect of i.p. administration of guava pomace extract (GPE) on a histamine- and serotonin-induced paw edema

**Groups**	**Dose (mg/kg)**	**Histamine (1 h)**		**Serotonin (1 h)**	
		**Mean Edema (∆V mL)**	**Percent inhibition**	**Mean Edema (∆V mL)**	**Percent inhibition**
		**1 h**		**1 h**	
Control		0.12 ± 0.02		0.07 ± 0.02	
Cyproheptadine	2	0.04 ± 0.02**	65	0.02 ± 0.02*	65
GPE	300	0.07 ± 0.02*	38	0.02 ± 0.02*	68
GPE	100	0.07 ± 0.01*	41	0.02 ± 0.01*	62
GPE	30	0.13 ± 0.01	-	0.03 ± 0.03	51

∆V = the values represent the mean difference of paw volume between the basal measure and other measure (1 h) ± S.E.M.; n = 5-6. *p < 0.05, **p < 0.01, significantly different when compared to control (ANOVA followed by Dunnett’s test).

**Figure 1:**
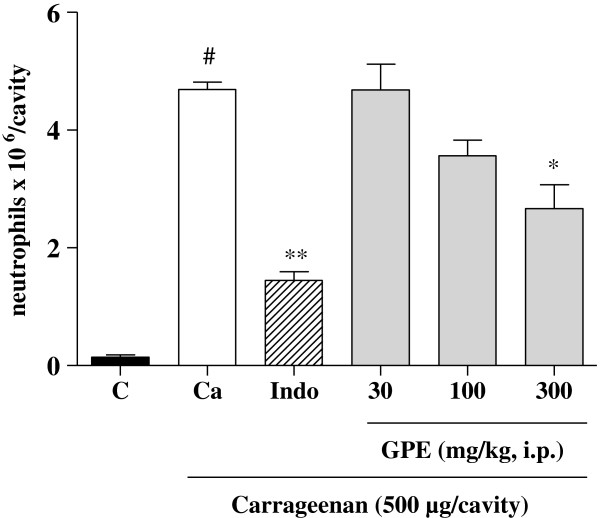
**Recruitment of neutrophils into the peritoneal cavity induced by carrageenan.** Control (C) treated with vehicle, Indomethacin (Indo) and Guava Pomace Extract (GPE) were followed by carrageenan injection. Mean ± S.E.M., n = 5-6. The symbol (#) indicates statistical difference compared to the vehicle group. The symbol (*) indicates statistical difference compared to the carrageenan group (ANOVA followed by Tukey’s test, *p < 0.05 and **p < 0.01).

As to the neutrophils migration in the peritoneal cavity model, the extract was able to inhibit in a significant (p < 0.05) and dose-dependent way, at the highest dose administered, the number of neutrophils into the peritoneal cavity induced by carrageenan (Figure 1). Thus, the extract was able to reduce edemas as well as inhibit the neutrophil migration quite significantly as evidenced by the anti-inflammatory activity. Therefore, the anti-inflammatory action of the guava pomace extract could be related to the inhibition of histamine, serotonin, bradykinin and prostaglandin, singly or in combination.

**Figure 2:**
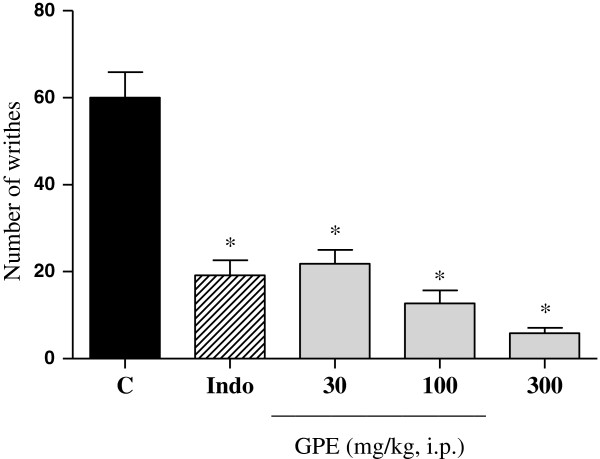
**Effect of i.p. injection of guava pomace extract on abdominal constriction induced by acetic acid in mice.** Control (C) treated with vehicle, Indomethacin (Indo) 10 mg/kg, Guava Pomace Extract (GPE). Mean ± S.E.M., n = 5-6. *p < 0.05 compared to the control group (ANOVA followed by Dunnett’s test).

**Figure 3:**
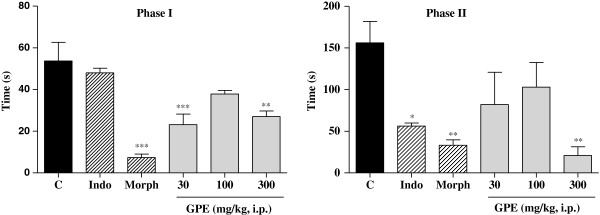
**Effect of i.p. injection of guava pomace extract on formalin-induced nociception in mice.** Control (C), Indo (Indomethacin 10 mg/kg), Morph (Morphine 10 mg/kg), Guava Pomace Extract (GPE). Mean ± S.E.M., n = 5-6. *p < 0.05, **p < 0.01 and ***p < 0.001 compared to the control group (ANOVA followed by Dunnett’s test).

The antinociceptive activity was initially evaluated by the acetic acid-induced abdominal writhing. The extract was able to inhibit in a significant (p < 0.05) and dose-dependent way the number of writhing induced by acetic acid (Figure 2). This unspecific test is still widely used in the evaluation of plant extracts that can act both centrally and peripherally, besides being considered a test for visceral inflammatory pain [13,14]. In order to proceed with the evaluation of the antinociceptive effect of the guava pomace extract, the formalin test was carried out. The administration of the extract at doses of 30 and 300 mg/kg (Figure 3) reduced the reaction time induced by formalin (p < 0.01 and p < 0.001), respectively in phase I (neurogenic phase), where the response is related with a direct activation of nociceptors. In phase II (inflammatory phase), marked by a local release of endogenous mediators, which generate a local inflammatory response [15], the extract was also active at the dose of 300 mg/kg (p < 0.01). Filho *et al.* demonstrated that quercetin was able to inhibit both phases of formalin-induced pain, with ID50 values of 374.10 mmol/kg and 103.00 mmol/kg for neurogenic and inflammatory phases, respectively [17]. Thus, extracts containing quercetin, such as GPE, could, therefore, also inhibit both phases in the formalin test.

From the chemical analysis, the present study found total phenolic values of 3.40 ± 0.09 mg GAE/g in the guava pomace extract. Literature data have shown in aqueous organic extracts of the pulp and peel portions of guava, estimated by the Folin-Ciocalteu’s method, values of 58.7 ± 4.0 and 26.3 ± 0.8 mg GAE/g, respectively [1]. Such difference between the values of total phenols detected in the fruit by Jiménez-Escrig *et al*. [1] and the pomace extract evaluated in the present study was already predictable, since the pressing process to which it was submitted in order to obtain the juice was able to remove most part of these compounds. Despite the low level of phenolic compounds present in the pomace, interesting results of the biological activity were demonstrated.

To determine the possible active compounds involved with the anti-inflammatory and antinociceptive effects of the pomace guava extract revealed in the present study, a GC/MS analysis was used, through which thirteen compounds were identified, among them epicatechin, quercetin, myricetin, isovanilic and gallic acids (Table 5).

**Table 5:** Phenolic compounds present in the guava pomace extract identified by GC-MS

***Peak number***	***Compound***	***RT (min)***	***% Area***	***Ions (m/z)***
1	Phosphoric acid, tristrimethylsilyl	5.789	2.39	299 (100), 73 (47), 300 (24), 314 (17), 301 (13); 341 (M+)
2	Beta-caryophyllene	6.978	0.65	41 (100), 93 (93), 133 (87), 91 (85), 69 (83); 189 (M+)
3	Malic acid (TMS)	7.219	0.73	73 (100), 147 (54), 233 (24), 245 (14), 133 (12); 335 (M+)
4	Alpha-selinene	7.460	0.59	93 (100), 107 (86), 189 (85), 91 (81), 41 (78), 205 (M+)
5	Trimethylsilyl 3-phenyl-2-propenoate	7.630	1.58	205 (100), 131 (87), 103 (67), 161 (62), 77 (48); 221 (M+)
11	Isovanilic acid	8.87	0.67	73 (100), 297 (67), 267 (48), 217 (42), 312 (40), 357 (M+)
16	Tris (trimethylsilyl) 2-[(trimethylsilyl)oxy]-1,2,3-propanetricarboxylate	9.176	3.69	73 (100), 273 (74), 147 (51), 217 (19), 347 (19); 467 (M+)
18	Beta-l-mannofuranose, 6-deoxy-1,2,3,5-tetrakis-o-(trimethylsilyl)	9.314	1.78	73 (100), 217 (96), 204 (42), 191 (23), 147 (22); 347 (M+)
19	Glucofuranoside, methyl 2,3,5,6-tetrakis-o-(trimethylsilyl)-, alpha-d-	9.353	2.79	217 (100), 73 (99), 218 (49), 129 (48), 191 (23); 363 (M+)
25	Gallic acid	9.89	3.72	73 (100), 281 (62), 458 (38), 332 (30), 147 (26); 464 (M+)
27	Hexadecanoic acid, trimethylsilyl ester	10.311	2.86	117 (100), 73 (79), 313 (74), 75 (58), 132 (52), 329 (M+)
31	Oleic acid, trimethylsilyl ester	11.425	1.51	73 (100), 117 (97), 75 (76), 129 (64), 339 (59), 354 (M+)
38	Epicatechin	17.41	0.61	368 (100), 73 (56), 355 (46), 369 (34), 650 (23), 654 (M+)
40	Myricetin	20.511	1.68	735 (100), 736 (66), 737 (39), 73 (36), 575 (33), 740 (M+)
41	Quercetin	20.601	4.94	647 (100), 648 (55), 649 (35), 73 (26), 650 (12), 663 (M+)

Peaks 6–11; 12–15; 17; 20–24; 28–30; 32–37 and 39 are not identified.

In general, the biological properties of guava have been already associated with its phenolic compounds, such as protocatechunic, ferulic, ascorbic, gallic and caffeic acids and quercetin [5,18]. In the present study, one of the identified compounds that could be responsible for the biological activity exhibited is the flavonoid quercetin, commonly known to have both antioxidant and anti-inflammatory effects, which inhibits NO and PGE_2_ activities. Furthermore the antinociceptive action of quercetin was also demonstrated through mechanisms that involve interaction with L-arginine-nitric oxide, serotonin, and GABAergic systems [19], moreover by inhibiting the pro-nociceptive cytokine production (e.g., TNF alpha and IL-1 beta) and the oxidative imbalance mediation of inflammatory pain [20].

A possible relationship between catechins and their involvement with the anti-inflammatory activity of the guava pomace extract can be compared with the immunomodulatory activity of black tea evaluated by Chattopadhyay *et al*., [20]. The major bioactive constituents of *Camellia sinensis* are catechins, which may have participated in the acute anti-inflammatory activity of the tea decoction evaluated using paw edema induced by carrageenan and dextran [20]. Many biological effects have been reported for (+) catechins, including anticarcinogen, cardiopreventive, antimicrobial, anti-viral, neuro-protective [20] and anti-inflammatory [21] effects. Similarly, the epicathechin identified in the guava pomace extract could be related with the anti-inflammatory effects showed in paw edema models induced by different phlogistic agents (Tables 1, 2, 3, 4) and neutrophils migration in the peritoneal cavity (Figure 1).

Finally, in relation to the anti-inflammatory and analgesic properties of guava, most studies refer to the leaf extracts and were evaluated on several experimental models [5,7,8]. In this paper, a promising anti-inflammatory and antinociceptive potential of the guava pomace extract was shown for the first time, despite the fact that its mechanism of action should be further investigated.

## Conclusions

Thus, we conclude that the presence of bioactive substances such as quercetin and epicatechin, as well as the important effects demonstrated in animal models, suggest that guava pomace could be a new source of compounds with anti-inflammatory and antinociceptive activities. Besides, applying this material in bioprocesses provides a wide range of alternative substrates, therefore, helping to solve pollution problems related to its disposal.

## Competing interests

The authors declare that they have no competing interest.

## Authors’ contributions

DC and FM were involved in the design of this study and performed pharmacological laboratory analyses and statistics. MP, MAP and BKB performed chemical laboratory analyses. de ASM and RPL drafted the manuscript along with the other authors. All authors read and approved the final manuscript.

## Pre-publication history

The pre-publication history for this paper can be accessed here:

http://www.biomedcentral.com/1472-6882/13/235/prepub
